# Is there a relationship between clinical stage and cardiovascular disease risk in bipolar disorder?

**DOI:** 10.1192/j.eurpsy.2022.1033

**Published:** 2022-09-01

**Authors:** K. Altınbaş, G. Kavak Sinanoğlu

**Affiliations:** Selçuk University, Psychiatry, Konya, Turkey

**Keywords:** clinical stage, cardiovascular disease risk, q risk3, bipolar disorder

## Abstract

**Introduction:**

Cardiovascular disease (CVD) is the leading cause of morbidity and mortality in bipolar disorders(BD). The heart age of patients with BD was found to be 8.5 years higher than gender-age matched health controls. Metabolic side effects of antipsychotics, poor diet, insufficient physical activity, smoking and sedentary life style increase the risk of cardiovascular disease in bipolar patients. QRISK-3 is an approved risk classification that calculates the 10-year risk of developing a heart attack or stroke.

**Objectives:**

This study aims to determine whether there is a difference between cardiovascular disease risk scores and clinical stages of bipolar disorder

**Methods:**

35 outpatients that were followed up in Selcuk University Medical Faculty were evaluated. The clinical stages and qrisk3 scores were calculated.

**Results:**

68.6% (n:24) of the patients were female. 42.9% of patients were in stage 3b (recurrent relapses, complete remission between episodes). The mean age was 36.94 ±10.46 years. The mean heart age was 50.54±17.35. The mean Q risk3 score was 5.59±8.18. There was no difference between bipolar patients at stage 2 and stage 3 in terms of age(p=0.36 and gender(p=0.73). When we compared the qrisk3 total socres and heart age of the patients in stage 2 and 3, we could not find any difference between groups (p=0.74, p=0.57 respectively).

**Conclusions:**

Even though we could not find any difference of qrisk scores at different clinical stages of patients with BD, the CVD risk increases with the age. Prospective longitudinal follow-up studies are required to evaluate dual interaction of clinical stages and CVD risk in BD.

**Disclosure:**

No significant relationships.

